# Tuum Est

**Published:** 1869-07

**Authors:** 


### Editorial Department.

### Tuum est.

The present number closes our eighth volume, and whatever may have been its character and worth, it is such as you have made it; according to our motto, *it is yours*. And since the Buffalo Medical and Surgical Journal belongs wholly to the profession, to its subscribers and contributors, I may be excused for offering congratulations upon this occasion, a period which marks its age, marks its progress, and, in some degree, marks its worth. Your Journal has passed the critical and tender age of infancy, and you all recollect what a fearful period in the history of this country gave it birth; it has come into years of full age, since it is the oldest medical journal in the State, and one of the oldest in the country. Age is a thing of which you may be proud; it is an evidence of natural vigor and soundness; it is also evidence of experience and worth; it is honorable, it shows strength. You have also made your Journal a successful one, as the world counts success; for it is a great success for a medical journal to be both alive and be paid for, and your Journal, for the most part, is paid for, that is, will be, when the bills to subscribers inclosed in this number are settled. You have made your Journal a progressive one, devoted wholly to rational medicine, not a "medicaster," but a reliable, earnest, firm, consistent supporter of the truth, recording faithfully all true progress in medicine and surgery, but not carried away with every wind of doctrine. In this respect you have made it a model worthy of imitation; nowhere can there be found a sheet which has been more truly "conservative" and more soundly progressive. It has received the sanction of the profession, and when this is obtained, nothing more is required to establish its character; it rests upon a rock.

You have made your Journal the history of the profession within the scope of its influence—of the whole profession. It contains a biographical history of the living medical men of Western New York and vicinity. The histories of some, it is true, being written, fully written, by nothing at all being said about them; their names not even appearing upon the subscription list. Your Journal is faithful in this respect; it is impartial. It photographs the whole family with painful exactness sometimes. I most heartily congratulate you in this respect, for coming generations of medical men will want to know who have lived before them, what were their names, their opinions, their practices, and you are furnishing the whole history, and giving it nearly "life size." For this purpose your medical journal is invaluable, and whatever of error it may contain in theory and practice, in surgery or obstetrics, here it is infallible—furnishes true histories of all living physicians, either with or without their consent. You have made your Journal faithful to the interests of medical men, to the progress and extension of medical science, and to the elevation and perfection of medical education. You have devoted its pages to independent, original thought, and to earnest practical wisdom. You have made it a journal for the medical student, the busy practitioner, and the mature medical philosopher.

This much for its past history; to you belongs the honor of its growth. Its future also rests in your hands; sure guarantee of usefulness. Your editor has made arrangements for the publication of your copy in much improved style and finish, so that volume nine may be expected to present an appearance never before attained. He would also return many thanks for the numerous tokens of favor and approval which he has received at your hands. Deeply sensible of the imperfect manner in which he has discharged his duty, these evidences of regard impress him more fully with a sacred obligation to serve you impartially and faithfully. To your keeping and care is confidently and trustingly committed the future welfare of the Journal. It is a "joint stock company," and all are invited to make personal effort to increase its capital, to extend its influence, and promote its usefulness; new subscribers thankfully received, and credit given for cash on old accounts.

